# LPS inmobilization on porous and non-porous supports as an approach for the isolation of anti-LPS host-defense peptides

**DOI:** 10.3389/fmicb.2013.00389

**Published:** 2013-12-17

**Authors:** Carlos López-Abarrategui, Alberto del Monte-Martínez, Osvaldo Reyes-Acosta, Octavio L. Franco, Anselmo J. Otero-González

**Affiliations:** ^1^Center for Protein Studies, Faculty of Biology, University of HavanaHavana, Cuba; ^2^Center for Genetic Engineering and BiotechnologyHavana, Cuba; ^3^Centro de Análises Proteômicas e Bioquímicas, Programa de Pós-Graduação em Ciências Genômicas e Biotecnologia, Universidade Católica de BrasíliaBrasília, Brazil

**Keywords:** LPS, antiendotoxic, antimicrobial peptides, affinity chromatography, LPS immobilization

## Abstract

Lipopolysaccharides (LPSs) are the major molecular component of the outer membrane of Gram-negative bacteria. This molecule is recognized as a sign of bacterial infection, responsible for the development of local inflammatory response and, in extreme cases, septic shock. Unfortunately, despite substantial advances in the pathophysiology of sepsis, there is no efficacious therapy against this syndrome yet. As a consequence, septic shock syndrome continues to increase, reaching mortality rates over 50% in some cases. Even though many preclinical studies and clinical trials have been conducted, there is no Food and Drug Administration-approved drug yet that interacts directly against LPS. Cationic host-defense peptides (HDPs) could be an alternative solution since they possess both antimicrobial and antiseptic properties. HDPs are small, positively charged peptides which are evolutionarily conserved components of the innate immune response. In fact, binding to diverse chemotypes of LPS and inhibition of LPS-induced pro-inflammatory cytokines from macrophages have been demonstrated for different HDPs. Curiously, none of them have been isolated by their affinity to LPS. A diversity of supports could be useful for such biological interaction and suitable for isolating HDPs that recognize LPS. This approach could expand the rational search for anti-LPS HDPs.

## INTRODUCTION

Sepsis is characterized by an uncontrolled inflammatory as well as anti-inflammatory process driven by the host immune system in response to bacteria (Adib-Conquy and Cavaillon, 2012). This syndrome is one of the leading causes of death in intensive care units worldwide and its incidence is progressively increasing (Kotsaki and Giamarellos-Bourboulis, 2012). Although major wall components of Gram-positive bacteria (peptidoglycan and lipoteichoc acid) can induce sepsis, the highest incidence of this syndrome is caused by lipopolysaccharides (LPSs) from Gram-negative bacteria (De Kimpe et al., 1995). Consequently, research in this field has been focused on LPS. LPSs are the major molecular component of the outer membrane of Gram-negative bacteria. This molecule represents a pathogen-associated molecular pattern (PAMP), responsible for the development of local inflammatory response through Toll-like receptor-4 (TLR-4) signaling (Miller et al., 2005). The inflammatory response is essential for bacterial clearence, but in extreme cases an exacerbated reaction may lead to septic shock (Salomao et al., 2012). Unfortunately, despite substantial advances in the pathophysiology of sepsis, there is no efficacious therapy against this syndrome yet (Schulte et al., 2013). As a consequence, septic shock syndrome continues to increase, reaching mortality rates over 50% in some cases (Buttenschoen et al., 2010).

In this context, the search for new therapeutics that can inhibit the activation of the innate immune system by LPS is of major importance (Pulido et al., 2012). Even though many studies in animal models and clinical trials have been conducted, there is no effective drug yet that interacts directly against LPS (Buttenschoen et al., 2010). Host-defense peptides (HDPs) could be a possible alternative solution since they possess antimicrobial, antiseptic, and immunomodulatory properties (Giuliani et al., 2010). These molecules have been identified as a defense strategy across many forms of life from prokaryotic organisms to vertebrates (Zasloff, 2002). HDPs are generally small, commonly having around 12–50 amino acid residues, cationic (net charge of +2 to +7), and are frequently quite hydrophobic and amphipathic (Jenssen et al., 2006). Furthermore, binding to diverse chemotypes of LPS and inhibition of LPS-induced pro-inflammatory cytokines from macrophages have been demonstrated for different HDPs (Scott et al., 2000; Lee et al., 2010). Interestingly, none of them have been isolated taking advantage of their affinity to LPS. As the search for new LPS-binding peptides is imperative for the development of more effective therapies, the use of LPS immobilized on different supports could be useful and suitable for isolating them. This approach could expand the rational search for anti-LPS HDPs.

## LIPOPOLYSACCHARIDE ENDOTOXIN

Lipopolysaccharides are the major molecular component of the outer membrane of Gram-negative bacteria. This molecule is essential for the survival of Gram-negative bacteria, contributing to the correct assembly of the outer membrane. In this context, LPS provides a permeability barrier to many different classes of molecules such as detergents, antibiotics, and metals. Due to their localization, LPS molecules participate in host-bacterium interactions like adhesion, colonization, virulence, and symbiosis (Silipo and Molinaro, 2011).

Lipopolysaccharide is an amphiphilic molecule composed of three domains: lipid A, core oligosaccharide, and O-antigen repeats. Lipid A represents the hydrophobic component of LPS, which is located in the outer leaflet of the outer membrane and carries the endotoxic properties of LPS. This domain is the most conserved region of the lipopolysaccharide molecule. The hydrophilic portion of the molecule is composed of the glycan, O-antigen. The core oligosaccharide joins the lipid A and O-antigen domains. The core oligosaccharide domain can be divided into two regions: the inner core (proximal to lipid A) and the outer core (proximal to O-antigen). In contrast to lipid A, core oligosaccharide and O-antigen domains are displayed on the surface of bacteria (Brandenburg et al., 2010).

The activation of the immune system by LPS occurs through the transmembrane protein TLR-4, a pattern recognition receptor (PRR) found on the surface of many cells from the innate immune system. MD-2, a small membrane-bound glycoprotein, associates with TLR-4 for the recognition of LPS. Other proteins such as CD14 and LPS-binding protein (LBP) enable the interaction of LPS with MD-2. After LPS recognition, TLR4 homodimerises, initiating the recruitment of intracellular adapter molecules such as MyD88, Mal, Trif, and Tram and leading to the expression of diverse inflammatory genes (Miller et al., 2005; Bryant et al., 2010; Mcgettrick and O’Neill, 2010).

## ANTI-LIPOPOLYSACCHARIDE HOST-DEFENSE PEPTIDES

The efficacy of HDPs against Gram-negative bacteria has been widely documented (Vaara, 2009; Cao et al., 2010; Park et al., 2011). The antimicrobial activity of these molecules is not only a consequence of their direct action against bacteria (at the cellular membrane and/or intracellular targets) but also of their anti-infective (modulation of the immune system) capacity (Hale and Hancock, 2007; Wieczorek et al., 2010). Although the outer membrane of Gram-negative bacteria constitutes an excellent permeability barrier to antibacterial agents, the interaction of HDPs with LPS permits this resistance mechanism to be bypassed. The process by HDPs across the outer membrane has been termed self-promoted uptake (Sawyer et al., 1988). In this mechanism, the peptides firstly interact with the negative surface of LPS and competitively displace the divalent cations that bridge the LPS barrier. This causes disturbance of the outer membrane, promoting peptide movement through it.

Host-defense peptides are very attractive molecules for use as therapeutics against septic syndrome due to their affinity for LPS and their antibacterial activity (Pulido et al., 2012). In fact, a number of natural HDPs from various sources bind to diverse chemotypes of LPS and reduce LPS-induced release of pro-inflammatory cytokines (Bowdish and Hancock, 2005; Bhattacharjya, 2010). For example, *in vitro* and *in vivo* LPS neutralization by the human cathelicidin peptide LL-37 has been demonstrated (Scott et al., 2002). The pretreatment of monocytes with this peptide inhibited *Pseudomonas aeruginosa* LPS-induced IL-8 production.

Interestingly, pro-inflammatory cytokine inhibition was abolished upon removal of LL-37 from the media before LPS stimulation, suggesting that the capacity of LL-37 to inhibit LPS signalling is dependent on extracellular LPS neutralization (Scott et al., 2011). Nevertheless, LL-37 may also have direct effects on macrophage function. Scott et al. (2002) demonstrated the upregulation of 29 genes and downregulation of another 20 genes in macrophages treated with the peptide using gene expression profiling experiments. Among the genes predicted to be up-regulated by LL-37 were those encoding chemokines and chemokine receptors, without stimulating the pro-inflammatory cytokine, TNF-α. Furthermore, an intracellular receptor for this peptide in monocytes has been discovered (Mookherjee et al., 2009). In order to increase the antiendotoxic activity of LL-37, various cathelicidin-derived peptides have been studied (Nagaoka et al., 2002; Nell et al., 2005). The antiendotoxic activity can be improved by increasing the hydrophobicity and cationicity of the parental peptide (Nagaoka et al., 2002). The LPS neutralization capacity of cathelicidins from another species has also been proved (Tossi et al., 1994; Tack et al., 2002; Bhunia et al., 2009).

Although the LPS neutralization properties of α-defensins are low (Scott et al., 2000), a potent antiendotoxic activity for some β-defensins has been established (Motzkus et al., 2006). Indeed, it has recently been demonstrated that LPS-binding activity and TNF-α release inhibition in RAW264.7 cultures for the human β-defensin DEFB114. Additionally, protection against LPS-induced reduction of human sperm motility *in vitro* and LPS-induced lethality of D-galactosamine-sensitized C57BL/6 mice were also demonstrated for DEFB114 (Yu et al., 2013). The antiendotoxic activity of DEFB114 was dependent on disulfide bond. On the other hand, fluorescence experiments demonstrated that DEFB126, another human β-defensin with anti-sepsis activity, can penetrate RAW 264.7 cells and diminish the production of LPS-stimulated inflammatory factors. In the same way, DEFB126 might also participate in intracellular immune regulation beyond its direct LPS neutralization (Liu et al., 2013). Perhaps DEFB126 uses a similar intracellular pathway to that of LL-37. Moreover, the differences in the antiendotoxic activity between α-defensins and β-defensins suggested that antibacterial activities do not necessarily correlate with anti-LPS properties (Bhattacharjya, 2010). Finally, the anti-LPS properties of invertebrate defensins have also been demonstrated (Saido-Sakanaka et al., 2004; Koyama et al., 2006). Other HDPs also have the capacity to inhibit LPS effects (Jacks et al., 1996; Giacometti et al., 2006; Lin et al., 2013; Schadich et al., 2013). These examples evidence the natural role of HDPs in the defense against LPS-induced septic shock.

Different mechanisms of LPS inhibition have been described for HDPs (Pulido et al., 2012). In general, the direct interaction of HDPs can disaggregate, or induce a change in the unilamellar/cubic structure of LPS to multilamellar, inhibiting the recognition of this molecule by the immune receptor complex (Andra et al., 2005; Kaconis et al., 2011; Singh et al., 2013). Besides, HDPs also can inhibit LPS-induced sepsis by their modulation of immune cells. In this context, the inhibition of pro-inflammatory mediators (Liu et al., 2013), inhibition of surface expression of TLR-4 by interacting with microtubules (Li et al., 2013), and the normalization of the coagulation (Kalle et al., 2012) have been demonstrated.

Otherwise, the structural knowledge of LPS-high affinity binders such as Limulus anti-LPS factor (Hoess et al., 1993), Bactericidal/permeability-increasing protein (Beamer et al., 1997), Factor C (Tan et al., 2000) and Polymyxin B (Pristovsek and Kidric, 1999) among others has allowed the development of synthetic antiendotoxic peptides (Pristovsek and Kidric, 2001; Andra et al., 2006; Ding et al., 2008). Although preclinical data are very encouraging, only one of the synthetic variants, the recombinant fragment of protein BPI (rBPI21), has been tested successfully in clinical studies (Domingues et al., 2012). The situation is similar for natural compounds where there is no Food and Drug Administration (FDA)-approved drug yet that interacts directly against LPS. Only the apheresis procedures for endotoxin adsorption with Polymyxin B (lipopeptide produced by the bacterium *Bacillus polymyxa*) immobilized in the fiber column have been used for the treatment of septic shock patients in Japan since 1994 (Ruberto et al., 2013). Due to the failure to obtain antiendotoxic molecules with clinical efficacy, searching for new LPS-binding peptides is imperative (Giuliani et al., 2010).

## LIPOPOLYSACCHARIDE IMMOBILIZATION

Affinity chromatography is one of the most efficient protein purification strategies. This technique is a method for selective purification of molecules from complex mixtures based on highly specific biological interaction between the immobilized ligand and the molecule of interest. The highly selective interactions that guide this procedure allow for a fast, often single-step process, with potential for purification in the order of several hundred to 1000-fold (Urh et al., 2009). Successful affinity purification is determined by the selection of a suitable support and immobilized ligand. The affinity support (the matrix onto which the ligand is immobilized) should selectively capture the molecule of interest while at the same time exhibiting low non-specific adsorption (Gustavsson and Larsson, 2006).

### ISOLATION OF LIPOPOLYSACCHARIDE-BINDING PROTEINS

Affinity supports based on LPS immobilization could be a powerful tool for the isolation of anti-LPS HDPs. Indeed, the isolation of LBPs using these supports has been described (Minetti et al., 1991; Chiou et al., 2000; Shahriar et al., 2006). For instance, the Limulus endotoxin-binding protein-protease inhibitor (LEBP-PI), a 12 kDa protein from Limulus amebocytes, was purified using an LPS affinity column. In this study, the authors immobilized LPSs onto Affi-Gel Hz support (Bio-Rad). This support is based on hydrazide coupling chemistry. Affi-Gel Hz hydrazide gel is an agarose support which reacts with the aldehydes of oxidized carbohydrates (periodate oxidation) to form stable, covalent hydrazone bonds (O’Shannessy and Wilchek, 1990). A high yield of active LEBP-PI was achieved after elution with LPS or sodium citrate (Minetti et al., 1991). On the other hand, Chiou et al. (2000) isolated an LBP by affinity chromatography based on LPS from *Escherichia coli* O55:B5 coupled to cyanogen bromide (CNBr) activated Sepharose CL-4B. The isolated glycoprotein showed an apparent molecular mass of about 40 kDa and 72.2% identity to tachylectin-3, a lectin isolated from the amebocyte of T. tridentatus, previously characterized by its affinity to the O-antigen of LPS. Shahriar et al. (2006) also used LPS-affinity chromatography for isolation of LBPs in porcine milk. The affinity support was prepared by coupling 100 mg of *E. coli* F4 LPS – 3 g of CNBr-activated Sepharose 4B. Low affinity LBPs were eluted using mild conditions (Tris 10 mM, 1 M NaCl, pH 7.2) whereas high affinity binders were eluted using 0.1 M glycine-HCl, pH 2.5. The LBPs lactoferrin, soluble CD14, serum amyloid A, alpha-S1 casein, beta-casein, and kappa-casein were isolated by this approach. The coupling reagent used in the last two examples for synthesizing the affinity support was CNBr, which is very efficient for immobilizing proteins. It activates hydroxyl groups on the resin to create reactive cyanate esters, which then can be coupled to amine-containing ligands forming an isourea bond (Urh et al., 2009). The LPS molecule is thus immobilized on these resins by their hexosamines located at the core outer region and in lipid A.

### ISOLATION OF LIPOPOLYSACCHARIDE-NEUTRALIZING PEPTIDES

Despite the success in purifying LBPs by affinity chromatography, this approach has not yet been used for isolating anti-LPS HDPs. The idea that interaction of LPS and anti-LPS peptides could be affected when one of them is immobilized on a matrix is possible, and therefore isolating anti-LPS peptides with immobilized LPS could be less efficient. Nevertheless, an interesting attempt to study LPS interaction with anti-LPS peptides when the latter were immobilized (Gustafsson et al., 2010) showed that immobilization of HDPs does not inhibit their capacity to neutralize LPS, although there are differences between the peptides assayed. The interaction of LPS with immobilized peptides was efficient both in LPS binding and inhibiting cytokine production induced by LPS.

**FIGURE 1:**
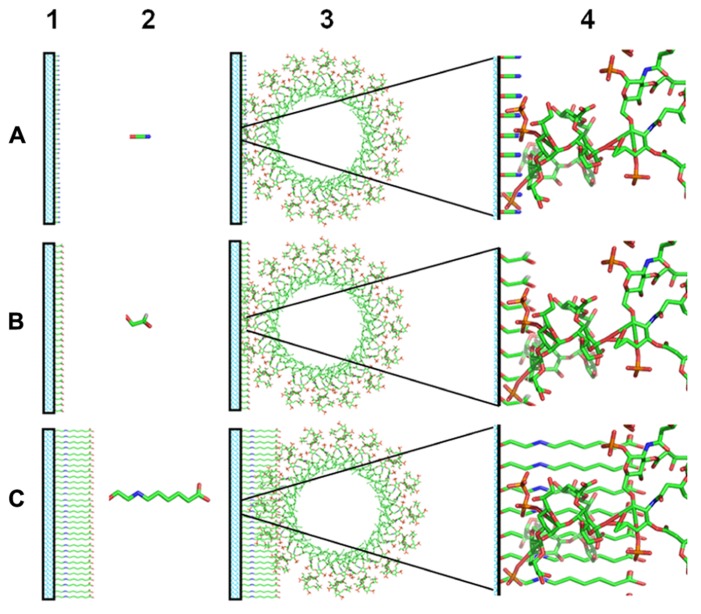
**Scheme of LPS immobilization on affinity supports.** (1) Activated supports. (2) Coupling reagents. (3) Miscellar lipid A immobilized on affinity supports. (4) Zoom of the interaction of lipid A with the activated supports. **(A)** Cyanogen bromide activated support. **(B)** Glyoxyl activated support. **(C)** Ethyloxy-6-aminocaproic acid activated support.

Otherwise, the binding of HDPs to LPS occurs through the lipid A moiety: specifically, basic aminoacids interact with phosphates and hydrophobic aminoacids with the acyl chains (Bhattacharjya et al., 2007). For this reason, the immobilization of LPS using hexosamines located in the lipid A region may affect recognition by HDPs. Therefore, a conjugation method keeping free the lipid A moiety in the lipopolysaccharide molecule could be more efficient to immobilize LPS for the isolation of anti-LPS HDPs. In fact, Pallarola and Battaglini (2008, 2009) performed the coupling of LPS with probes bearing hydrazine or primary amino groups. LPS was modified through the activation of the hydroxyl groups present in its O-antigen moiety. Conjugates with good labeling ratios were obtained with the preservation of its endotoxic activity. A similar strategy was discussed above for the purification of LEBP-PI (Minetti et al., 1991). Otherwise, LPS molecules are capable of form micelles and aggregates even at very low concentrations (Yu et al., 2006). For this reason, the efficiency of LPS immobilization could be extremely low. Unfortunately, there is no data reported about the efficiency of LPS immobilization. Moreover, the coupling of LPS through the O-antigen domain may render a higher immobilization efficiency than coupling LPS through hexosamines due to the poor reactivity of the latter (Eller et al., 2000). Nevertheless, the LPS immobilization through hexosamines could not be discarded due the large numbers of commercial resins that could be activated to react with amine groups (Urh et al., 2009). In this sense, the accurate use of coupling reagents may influences on the LPS affinity support synthesis. For example, coupling reagents with a higher length seems to better interact with LPS (**Figure 1**).

On the other hand, magnetic field-based separations using magnetic nanoparticles have received considerable attention in the last two decades (Horak et al., 2007). This methodology can be used on viscous materials, simplifying the purification process by removing sample pretreatment. Furthermore, the highly specific surface area of the nanoparticles enables the immobilization of a larger amount of molecule. Therefore, the immobilization of LPS on magnetic nanoparticles could be a very attractive procedure for isolating anti-LPS HDPs by magnetic separation. Although this approach has not been used yet, there are different examples of LPS immobilization on magnetic nanoparticles (Fornara et al., 2008; Piazza et al., 2011). For example, LPS was reversibly immobilized in a magnetic nanoparticle system consisting of oleylamine-coated iron oxide nanocrystals by hydrophobic interactions. LPS-magnetic nanoparticles were stable enough to mimic natural LPS aggregates for investigating the interaction of the LPS with TLR4 receptor (Piazza et al., 2011). In another approach, Fornara et al. (2008) synthesized magnetic nanoparticles for detection of *Brucella* antibodies in biological samples. Thermally blocked nanoparticles obtained by thermal hydrolysis were functionalized with LPS from *Brucella abortus*. LPS was attached to magnetic nanoparticles by adsorption through hydrophobic interactions, and the variation in magnetic relaxation due to surface binding of antibodies to LPS-functionalized nanoparticles was used to detect the disease. This method showed high sensitivity, with detection limit of 0.05 μg/mL of antibody in the biological samples without any pretreatment. Interestingly, the same approach could be used for detecting anti-LBPs from different sources. The same principle is not feasible for HDPs due to their small molecular weight. Furthermore, both systems described above could be used as affinity purification procedures, but the stability of hydrophobic LPS-functionalized nanoparticles in drastic elution conditions (0.1 M glycine-HCl, pH 2.5) would have to be evaluated.

As was exemplified above, the synthesis of affinity supports is no longer used only for purification of specific biomolecules. It is also rapidly becoming a method of choice to study biological interactions. In fact, Genfa et al. (2005) identified LPS-binding molecules in herb fractions by coating affinity optical biosensor cuvettes with lipid A via hydrophobic interactions after pre-incubating extracts with LPS. Such a concept demonstrated that LPS can be immobilized, keeping its ability to efficiently bind LPS binding molecules, and it could represent a high-throughput approach for the identification of LPS-neutralizing peptides (Zheng et al., 2010).

## CONCLUDING REMARKS

Despite substantial advances in the pathophysiology of sepsis, there is no effective therapy against this syndrome yet. A therapeutic alternative could be the use of HDPs due to the capacity of some of them to neutralize LPS. As it is vital to find new LPS-binding peptides for the development of more effective therapies, the use of LPS immobilized on different supports could be useful and suitable for isolating them. This approach could expand the rational search for anti-LPS HDPs.

## Conflict of Interest Statement

The authors declare that the research was conducted in the absence of any commercial or financial relationships that could be construed as a potential conflict of interest.
